# Mapping out the gut microbiota-dependent trimethylamine N-oxide super pathway for systems biology applications

**DOI:** 10.3389/fsysb.2023.1074749

**Published:** 2023-03-08

**Authors:** Isabel M. E. Valenbreder, Sonia Balăn, Marian Breuer, Michiel E. Adriaens

**Affiliations:** Maastricht Centre for Systems Biology (MaCSBio), Maastricht University, Maastricht, Netherlands

**Keywords:** gut microbiome, TMA/TMAO pathway, constraint-based modeling, gut-heart axis, pathway, whole-body metabolic model, heart failure

## Abstract

The metabolic axis linking the gut microbiome and heart is increasingly being researched in the context of cardiovascular health. The gut microbiota-derived trimethylamine/trimethylamine N-oxide (TMA/TMAO) pathway is responsible along this axis for the bioconversion of dietary precursors into TMA/TMAO and has been implicated in the progression of heart failure and dysbiosis through a positive-feedback interaction. Systems biology approaches in the context of researching this interaction offer an additional dimension for deepening the understanding of metabolism along the gut-heart axis. For instance, genome-scale metabolic models allow to study the functional role of pathways of interest in the context of an entire cellular or even whole-body metabolic network. In this mini review, we provide an overview of the latest findings on the TMA/TMAO super pathway and summarize the current state of knowledge in a curated pathway map on the community platform WikiPathways. The pathway map can serve both as a starting point for continual curation by the community as well as a resource for systems biology modeling studies. This has many applications, including addressing remaining gaps in our understanding of the gut-heart axis. We discuss how the curated pathway can inform a further curation and implementation of the pathway in existing whole-body metabolic models, which will allow researchers to computationally simulate this pathway to further understand its role in cardiovascular metabolism.

## 1 Introduction

### 1.1 Crosstalk along the gut-heart axis

A better understanding of the gut-heart axis can improve our understanding of the metabolic progression of cardiovascular diseases (Kazemian et al., 2020; Salzano et al., 2020). At one end of the axis, human cardiac health has been shown to influence the metabolic activity of the gut microbiome as a significant reduction in cardiac output forces changes in the host’s intestinal functions (Trøseid et al., 2020; Aaron et al., 2021; Zhang et al., 2021). At the other end of the axis, the gut microbiome affects cardiovascular health by synthesizing beneficial metabolites from dietary fibers (Vacca et al., 2020; Coutinho-Wolino et al., 2021), including short chain fatty acids, bile acids, and amino acids (Tuerhongjiang et al., 2021). These metabolites are involved in immune modulation and inflammation regulation by ensuring the integrity of the intestinal mucosal barrier (Trøseid et al., 2020), thereby minimizing the entry of endotoxins into the circulation (Vancamelbeke and Vermeire, 2017). There is increasing evidence of a positive feedback loop along the gut-heart axis resulting from dysbiosis and heart failure (Nagatomo and Tang, 2015; Kitai et al., 2016; Feng et al., 2021).

Trimethylamine N-oxide (TMAO) is a gut microbiota-derived metabolite which has been identified as a potential diagnostic and prognostic marker for heart failure. The overabundance of TMAO and its precursor trimethylamine (TMA) in the systemic blood circulation has been linked to several perturbations of the organism’s metabolism such as platelet hyperactivity and inflammatory responses (Trøseid et al., 2020). This can lead to the development of coronary artery disease, myocardial hypertrophy, and the progression of heart failure (Jie et al., 2017; Toya et al., 2020). The TMA/TMAO metabolic pathway thus constitutes an important metabolic link between the gut microbiome and diseased heart.

### 1.2 Systems biology approaches to the gut-heart axis

Systems biology approaches have the potential to predict the behavior of complex systems *via* computationally simulated conditions, thereby reducing the need for expensive and invasive procedures on human patients (Hyduke et al., 2013). In the domain of metabolism, one of the most powerful systems biology approaches is genome-scale metabolic modeling. Genome-scale metabolic models (GEMs) allow the functional role of pathways belonging to an entire cellular or even whole-body metabolic network to be studied. The models represent the reactions in a given pathway by a stoichiometric matrix and are able to balance the total input and output of every compound to be in steady state (Orth et al., 2010).

Lower and upper bounds can be given to any reaction, limiting the minimal and maximal fluxes that are allowed to pass through reactions along the pathway; such bounds are applied to mimic a certain tissue, person and/or condition (ex. nutrient uptake rates). Whole-body metabolic models (WBMs) are a subclass of GEMs that contain compartments that mimic different organs, including the heart and the microbiome in the gut. WBMs thereby allow us to study interactions along the gut-heart axis. The first male and female WBMs were recently published by Thiele, Sahoo (Thiele et al., 2020). These models include sex-specific and sex-nonspecific organs, biofluid compartments, and curated metabolite exchange reactions between them. These WBMs are freely available in an SBML format which means they can be used across platforms and languages. The models are additionally native to the constraint-based metabolic modeling (COBRA) toolbox (Heirendt et al., 2019), which allows for simulation and analysis but also further model refinement and expansion.

In the case of the TMA/TMAO super pathway, systems biology approaches offer an additional dimension for deepening the understanding of metabolism along the gut-heart axis (Huttenhower et al., 2012; Magnúsdóttir and Thiele, 2018). For example, WBMs could allow one to simulate changes along the gut-heart axis by changing relevant reaction constraints. However, the conversion of metabolites through the TMA/TMAO pathway with respect to the gut-heart axis has only been visualized in a limited number of publications (Tang et al., 2013; Koeth et al., 2014; Fennema et al., 2016; Janeiro et al., 2018), and has not yet been curated for modeling purposes. Consequently, our aim was to summarize the current state of gut-heart axis knowledge in a curated pathway, that can serve as a resource for continued community-based curation and for systems biology modeling studies to further our understanding of the gut-heart axis.

## 2 Drafting the pathway: A metabolite-by-metabolite approach

In order to curate the TMA/TMAO pathway for WikiPathways, we first reviewed current literature on each of the TMA/TMAO pathway metabolites in the context of heart failure using relevant Pubmed and Google Scholar search terms (TMA, TMAO, heart failure, and gut microbiome). Concurrently, the male WBM (dubbed Harvey) (Thiele et al., 2020) was used for an initial exploration of the extent to which the TMA/TMAO pathway and its links to the gut-heart axis are currently already mapped in a body-scale metabolic network. We limited the scope of this exploration to the male model under the assumption that findings in literature would be biased towards the male physiology, especially in cases where the sex of the subjects is not mentioned. Within the model, we searched for ‘tma’, ‘tmao’, and their precursors ‘choline’, ‘betaine’, and ‘L-carnitine’ using the COBRA Toolbox (Heirendt et al., 2019). While certain reaction pathways were present in the Harvey implementation of the TMA/TMAO pathway and outlined in the literature search, there were also reaction pathways not accounted for in the model including most of the conversion steps from dietary precursors to TMA. A survey of existing literature was then performed, to find additional information on these particular missing reactions and their mechanisms *via* a similar approach, with refined search queries (ex. ‘betaine’,‘conversion’, ‘pathway’).

In the next sections, the individual pathways of each of the metabolites will be outlined and discussed in the context of relevant literature and the Harvey WBM.

### 2.1 Choline

While humans can endogenously produce choline *via* the hepatic phosphatidylethanolamine N-methyltransferase pathway, additional dietary choline ingestion is required to meet the physiological demand (Arias et al., 2020). The direct metabolism of free choline from dietary sources is mediated by sodium-independent carriers in the small intestine (Cho et al., 2020). Free choline can then be metabolized to either acetylcholine, betaine, phospholipids, or TMA (Arias et al., 2020). Prior to absorption into the portal circulation, dietary choline is transformed into TMA in the intestinal lumen by members of the Proteobacteria and Firmicutes phyla (Cho et al., 2020). This transformation is believed to be mediated by two distinct choline utilization (Cut) gene clusters, CutC and CutD, which are responsible for encoding a glycyl radical enzyme and a glycyl radical activating protein, respectively (Craciun and Balskus, 2012; Bodea and Balskus, 2018).

Choline metabolism in the Harvey WBM model begins when choline is partially absorbed into the cytosol of the stomach cells (*Stomach_chol[c]*) after which it is distributed into the blood circulation (*chol[bc]*). It then travels to the kidneys (*Kidney_chol[e]*), where it enters the renal blood circulation (*Kidney_chol[bcK]*), is absorbed into the cytosol (*Kidney_chol[c]*), and finally filtered and excreted in the urine (*chol[u]*). The choline that does not get absorbed in the stomach stays in the luminal compartment of the GI tract (*chol[lu]*) and reaches the small intestine. The choline conversion reaction into TMA is missing in the Harvey WBM.

### 2.2 Betaine

Betaine is an amino acid derivative endogenously produced in many living organisms (Koistinen et al., 2019). It plays an important role in diverse metabolic processes due to its methyl-donating capacities (Eklund et al., 2005). Although betaine is an important nutrient and precursor of other metabolites, including TMA/TMAO, research on the betaine pathway and metabolism is limited. There is a dynamic trade-off between betaine and TMA levels, in which the production level of choline determines whether betaine contributes to TMA/TMAO synthesis. Choline is a metabolite that can be transformed into either TMA or betaine. Increased betaine production can therefore result in reduced overall levels of TMA (Ebert et al., 2020). High choline production rates, however, can contribute to increased levels of both betaine and TMA (Ebert et al., 2020). A specific microbial community is responsible for the synthesis and subsequent uptake of betaine from choline in the intestinal lumen (Zhao et al., 2018). This occurs in a two-step oxidation process of choline, which is catalyzed by choline dehydrogenase and subsequently betaine-aldehyde dehydrogenase (Gadda, 2020). After its synthesis, betaine is absorbed by enterocytes and distributed *via* the portal vein to other tissues for hepatic and renal catabolism (Craig, 2004). Betaine is hypothesized to contribute to TMA/TMAO formation both directly and indirectly. The direct conversion of betaine to TMA is catalyzed by betaine reductase *via* a Stickland fermentation mechanism (Naumann et al., 1983; Fennema et al., 2016). Alternatively, betaine can undergo demethylation to form an intermediate dimethylglycine molecule, which can form TMA *via* decarboxylation (Wood et al., 2010). Notably, the rate of the dietary betaine to TMA conversion *in vitro* happens at a much lower rate than the direct transformation of choline into TMA, and there is no current evidence to support a direct conversion mechanism taking place *in vivo* (Wang et al., 2014).

The exogenous betaine (identifier in model: *glyb[d]*) within the WBM model is distributed in the intestinal lumen (*glyb[lu]*), after which it is is assumed to be enzymatically converted, as the reaction was not found in present in the model into TMA and absorbed into the intestinal endothelium. The remaining betaine is excreted in the feces (*glyb[fe]*). Endogenous betaine (see Supplementary Figure S1) in the blood circulation (*glyb[bc]*) is distributed to the liver (*Liver_glyb[e]*) and kidneys (*Kidney_glyb[e]*), the latter being the organ where it is filtered and excreted in the urine (*glyb[u]*). No reaction was found to account for the dependence of betaine levels on choline availability. As with choline, the conversion step of betaine to TMA was absent in the model.

### 2.3 L-carnitine

L-carnitine must also be supplemented *via* the host’s dietary intake as its endogenous production is not sufficient for supporting cellular function (Evans and Fornasini, 2003). The microbial transformation of L-carnitine into TMA occurs partially *via* the L-carnitine monooxygenases CntA and CntB (Quareshy et al., 2021). This reaction mechanism requires molecular oxygen as a cofactor and is therefore limited by the anoxic conditions in the intestinal lumen (Rajakovich et al., 2021). Rajakovich, Fu (Rajakovich et al., 2021) have since proposed a second reaction mechanism for this conversion in which oral L-carnitine ingestion forms an intermediate metabolite, gamma-butyrobetaine (γBB) (Rajakovich et al., 2021). Dietary supplementation of γBB was demonstrated to significantly increase TMA and TMAO in anaerobic bacterial cultures (Rajakovich et al., 2021). A more recent discovery in RNA-sequencing analyses found that the subsequent conversion of γBB to TMA is mediated by the bbu gene cluster (Rajakovich et al., 2021; Buffa et al., 2022). Hence, γBB is a major product of gut microbiota-dependent catabolism of dietary L-carnitine and an important intermediate in the TMA/TMAO pathway.

Within the Harvey WBM model, exogenous L-carnitine (*crn[d]*) directly reaches the lumen of the GI system (*crn[lu]*) after ingestion. There is no reaction currently present that would indicate microbial conversion into TMA *via* γBB. This conversion was nonetheless added in the visualization of the pathway as there is sufficient biological evidence for its occurrence. The unabsorbed L-carnitine from the intestinal lumen is excreted in feces (*crn[fe]*). Endogenous L-carnitine is found in the blood circulation (*crn[bc]*) and distributed to the liver (*Liver_crn[e]*), where it is absorbed into the cells (*Liver_crn[c]*) as shown in Supplementary Figure S2. Endogenous L-carnitine is also absorbed into the colonocytes (*Colon_crn[c]*). Finally, the reactions show the presence of L-carnitine in the blood circulation (*Kidney_crn[bcK]*), extracellular space (*Kidney_crn[e]*) and cytosol of the kidneys (*Kidney_crn[c]*), from where it is filtered and excreted through the urine. No reaction was found to metabolize l-carnitine to TMA.

### 2.4 TMA and TMAO

After the microbial derivation from its precursors, TMA is readily absorbed across the enterocytes and enters the portal circulation. It is then absorbed by the hepatocytes where flavin-dependent monooxygenase 3 (FMO3) catalyzes the conversion of TMA into TMAO (Fennema et al., 2016). Existing literature clearly establishes the direct link between TMA, FMO3, TMAO and the emergence of heart failure (Obeid et al., 2016; Tang et al., 2019). As TMA is a product of microbial conversion of dietary nutrients, FMO3 illustrates an important functional example of host-gut microbiome metabolic interactions in relation to super pathways of the gut-heart axis (Nicholson Jeremy et al., 2012). However, it is important to highlight that the amount of TMAO entering the systemic circulation to reach the heart depends on the various interindividual and genetic factors affecting dietary precursor availability, gut microbiome composition, and microbial gut action to first produce TMA (Fennema et al., 2016).

TMA metabolism within the Harvey WBM model was observed to start in the lumen of the small intestine (*tma[luSI]*) after its successful microbial conversion from dietary precursors. As previously mentioned, these conversion steps are present in our visualization but absent from the Harvey WBM model. TMA is distributed to the lumen of the large intestine (*tma[lull]*) and absorbed across the apical membrane of colonocytes (*Colon_tma[c]*). The TMAO that directly comes from the diet (*tmao[d]*) is distributed to the lumen of the GI tract, where it is converted by the actions of TMAO reductase into TMA. Here, the two pathways coming from microbial TMA production and dietary TMAO to TMA reduction converge in the absorption of TMA into colonocytes (*Colon_tma[luC]*). From there, TMA enters the vascular system of the colon (*Colon_tma[bpC]*) until it finally enters the portal circulation (*tma[bp]*). TMA is further carried to the liver, where it is absorbed into the hepatic cytosol (*Liver_tma[c]*). At this stage, the metabolic pathway splits again. A proportion of the TMA that has been absorbed into the liver is oxidized into TMAO by FMO3 (*Liver_tmao[c]*). After intracellular conversion, TMAO enters the extracellular space of the liver (*Liver_tmao[e]*) to be distributed throughout the systemic blood circulation (*tmao[bc]*). TMA that is not oxidized is excreted from the liver and enters the blood circulation, before being filtered by the kidneys (*Kidney_tma[bcK]*) and urinarily excreted (*tma[u]*). Similarly, the body regulates TMAO concentrations by plasma TMAO filtration (*Kidney_tmao[bcK]*) and subsequent urinary excretion (*tmao[u]*). There is no explicit reaction defined for the transport of TMAO by the heart compartment after entering the host’s systemic blood circulation.

### 2.5 Reflecting on the harvey WBM

**FIGURE 1:**
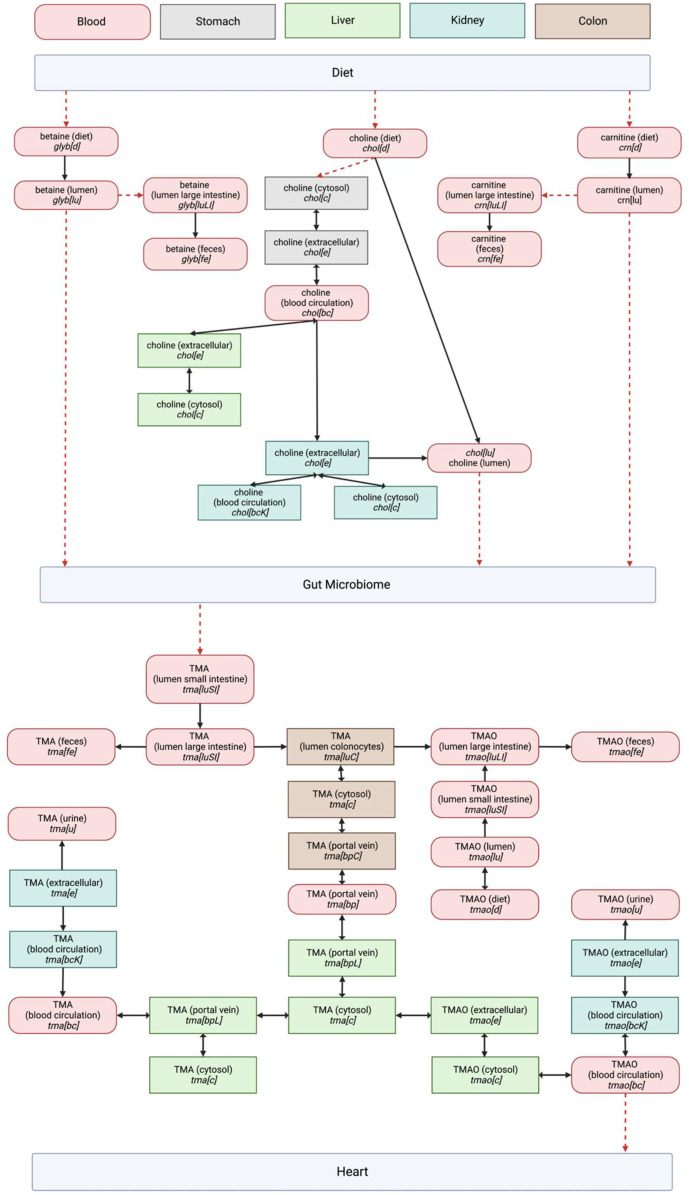
Entire TMA/TMAO pathway visualization in the Harvey model including dietary precursors and microbial metabolism. Organ compartments are color coded as indicated in the legend at the top of the page. The abbreviations used for metabolite compartments followed those in Thiele, Sahoo (Kanehisa and Goto, 2000): where bc = Blood, circulation, bcK = Blood, circulation (kidney), bp = Blood, portal vein, bpL = Blood, portal vein (liver), c = cytosol, d = diet, e = Extracellular, fe = Feces, lu = Lumen, luLI = Lumen, large intestine, luSI = Lumen, small intestine, u = Urine. The directionality of reactions as presented in Harvey is represented by single- or double-headed arrows. Red dashed arrows represent reactions that were expected based on our literature study, but not observed in the Harvey WBM model.

A survey of the existing reactions within the Harvey WBM model involved in the TMA/TMAO pathway was successfully visualized into a network to show their distribution between the gut microbiome and heart compartments (Figure 1).

We retained the WBM’s reaction metabolite IDs, which describe the cellular compartments for individual organs to enable an easier integration of a future pathway curation back into the WBM (Thiele et al., 2020). The biological relevance of each pathway component was evaluated using literature, revealing a significant overlap between the expected biological network and the pathway structure in the Harvey WBM. The model is missing reactions for the conversion of dietary TMA precursors into TMA, and the subsequent transport of TMAO through the host’s systemic system to reach the heart. Because these fundamental connections of the pathway to the WBM’s dietary and heart compartments are lacking, using the model to observe changes along the gut-heart axis due to TMA/TMAO metabolism is currently out of scope.

## 3 Pathway curation

**FIGURE 2:**
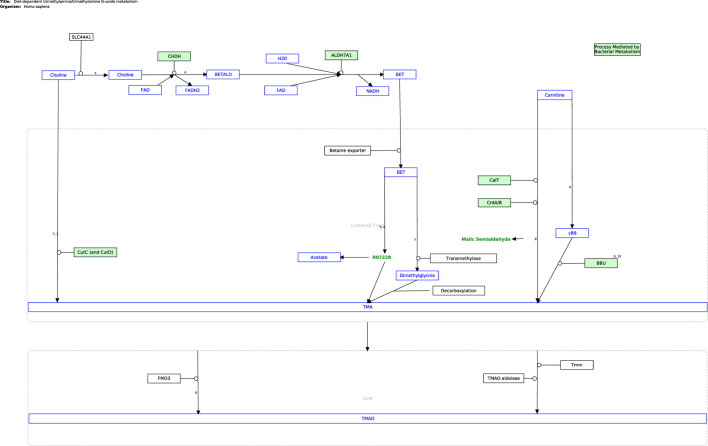
The curated TMA/TMAO pathway produced using PathVisio. The pathway is also available on the Classic WikiPathways at database https://classic.wikipathways.org/index.php/Pathway:WP5219.

The next step in the curation process was to cross-check the information from published literature with related pathway content from open-source repositories (KEGG (Kanehisa and Goto, 2000), Reactome (Gillespie et al., 2022), and WikiPathways (Kelder et al., 2009)), as presented in Supplementary Table S1. Currently, the only established pathway relating directly to the TMA/TMAO pathway is the metabolic conversion of choline into betaine in the Choline Catabolism pathway in Reactome (Choline Catabolism, 2015), which was considered in curating the TMA/TMAO pathway. WikiPathways was selected as the platform for curation as it is an open-based resource that allows the scientific community to update and elaborate on published pathways, as well as use them for functional analysis and data integration using systems biology tools (Martens et al., 2020). The Diet-Dependent Trimethylamine/Trimethylamine N-Oxide Metabolism pathway (Figure 2) was constructed in PathVisio and subsequently published on Wikipathways https://www.wikipathways.org/index.php/Pathway:WP5219.

## 4 Discussion

The curation of the TMA/TMAO pathway has the potential to enable the development of an accurate reflection of the gut-heart axis through community-based iterations. To conclude this exploration, we will discuss the current and future applications of our curated pathway.

The curated TMA/TMAO pathway encapsulates the currently known biochemistry of TMA/TMAO metabolism between the diet, gastrointestinal tract, and heart. In its current state in GPML format, the pathway is available for pathway enrichment analysis and data visualization using -omics data sets in popular pathway analysis tools like PathVisio (Kutmon et al., 2015).

Future discoveries by the scientific community which improve our understanding of the TMA/TMAO pathway can be readily incorporated by adapting the pathway on WikiPathways accordingly (Adriaens et al., 2008). Furthermore, the pathway has been curated with an eye on implementation in the Harvey WBM. Thus, all required information is present in the visualization in Figure 1 and the WikiPathways would be required for this implementation. Expanding the Harvey WBM in this way is beyond the scope of the curation of the pathway itself presented in this article but would allow to leverage whole-body constraint-based metabolic modeling in the further analysis of the pathway in the context of the gut-heart axis. For example, by changing heart-, diet-, or microbial composition-constraints provided in the COBRA toolbox, one could observe the resulting effect along the gut-heart axis *via* flux-based or flux variance analyses. These systems biology approaches have been demonstrated to be successful in researching the gut-brain axis (Thiele et al., 2020; Mohammad et al., 2022). For example, Mohammad, Palukuri (Mohammad et al., 2022), constrained the gut microbiome with different dietary constraints to analyze its effect along the gut-brain axis in autistic *versus* non-autistic hosts (Mohammad et al., 2022).

## 5 Concluding remarks

By using WikiPathways, it will be possible to maintain an accurate visual representation of the TMA/TMAO pathway with each community-based iteration. There are still gaps in our current understanding of the pathway, including the conversion of betaine directly into TMA, which has only recently been observed to occur (Wang et al., 2014) or the exact relative contributions of dietary *versus* endogenous precursors in TMA production. One mitigating approach to filling these gaps is applying a constraint-based modeling approach in combination with metabolomics data to increase the accuracy and coverage of the pathway by identifying missing reactions (Haas et al., 2022). There are several metabolomics datasets available for both cardiac and gut microbial activity relevant for this purpose (Fromentin et al., 2022; Talmor-Barkan et al., 2022). The pathway’s current form on Wikipathways encourages the scientific community to take part in the continued improvement and usage of this pathway for modeling purposes.

## Supplementary material

SUPPLEMENTARY FIGURE S1 Endogenous precursor pathway for betaine in the Harvey WBM. Abbreviations of metabolite compartments based on Thiele, Sahoo (20). Double arrowed connection represents a flux of metabolite in both directions whereas a unidirectional arrow represents the flux in one direction. Dashed arrows represent reactions that were not observed in the Harvey WBM model.

SUPPLEMENTARY FIGURE 2 Endogenous precursor pathway for l-carnitine in the Harvey WBM. Abbreviations of metabolite compartments based on Thiele, Sahoo (20). Double arrowed connection represents a flux of metabolite in both directions whereas a unidirectional arrow represents the flux in one direction. Dashed arrows represent reactions that were not observed in the Harvey WBM model.
