# Supplementary material for: Mapping out the gut microbiota-dependent trimethylamine N-oxide super pathway for systems biology applications
Source: Front Syst Biol. 2023 Mar 8;3:1074749. doi: 10.3389/fsysb.2023.1074749 (PMC12342028; doi:10.3389/fsysb.2023.1074749)
Supplement: Supplementary file 1 [file Table1.DOCX]

**Supplementary Table 1.** An overview of published pathway titles from the KEGG, Reactome, BIGG, and Wikipathways repositories in which the defined TMA/TMAO pathway metabolites are present.

| **Metabolite** | **KEGG pathways** | **Reactome pathways** | **Wikipathways pathways** |
| --- | --- | --- | --- |
| Betaine | Glycine, serine and threonine metabolism  Metabolic pathways  ABC transporters | Choline catabolism  Sulfur amino acid metabolism | Choline catabolism |
| Choline | Glycine, serine and threonine metabolism  Teichoic acid biosynthesis Glycerophospholipid metabolism  Metabolic pathways  ABC transporters  Cholinergic synapse  Bile secretion  Choline metabolism in cancer | Choline catabolism | Choline catabolism |
| L-carnitine | Thermogenesis  Bile secretion  Diabetic cardiomyopathy | Carnitine synthesis  Carnitine metabolism |  |
| TMA | Methane metabolism  Metabolic pathways  Microbial metabolism in diverse environments  Carbon metabolism | Defective FMO3 causes TMAU |  |
| TMAO | Methane metabolism  Metabolic pathways  Microbial metabolism in diverse environments  Two-component system | FMO oxidizes nucleophiles (Homo sapiens) | Nicotine metabolism in liver cells  Cocaine metabolism  Tamoxifen metabolism  1q21.1 Copy number variation syndrome |
