# Supplementary figures and images for: Mapping out the gut microbiota-dependent trimethylamine N-oxide super pathway for systems biology applications
Source: Front Syst Biol. 2023 Mar 8;3:1074749. doi: 10.3389/fsysb.2023.1074749 (PMC12342028; doi:10.3389/fsysb.2023.1074749)

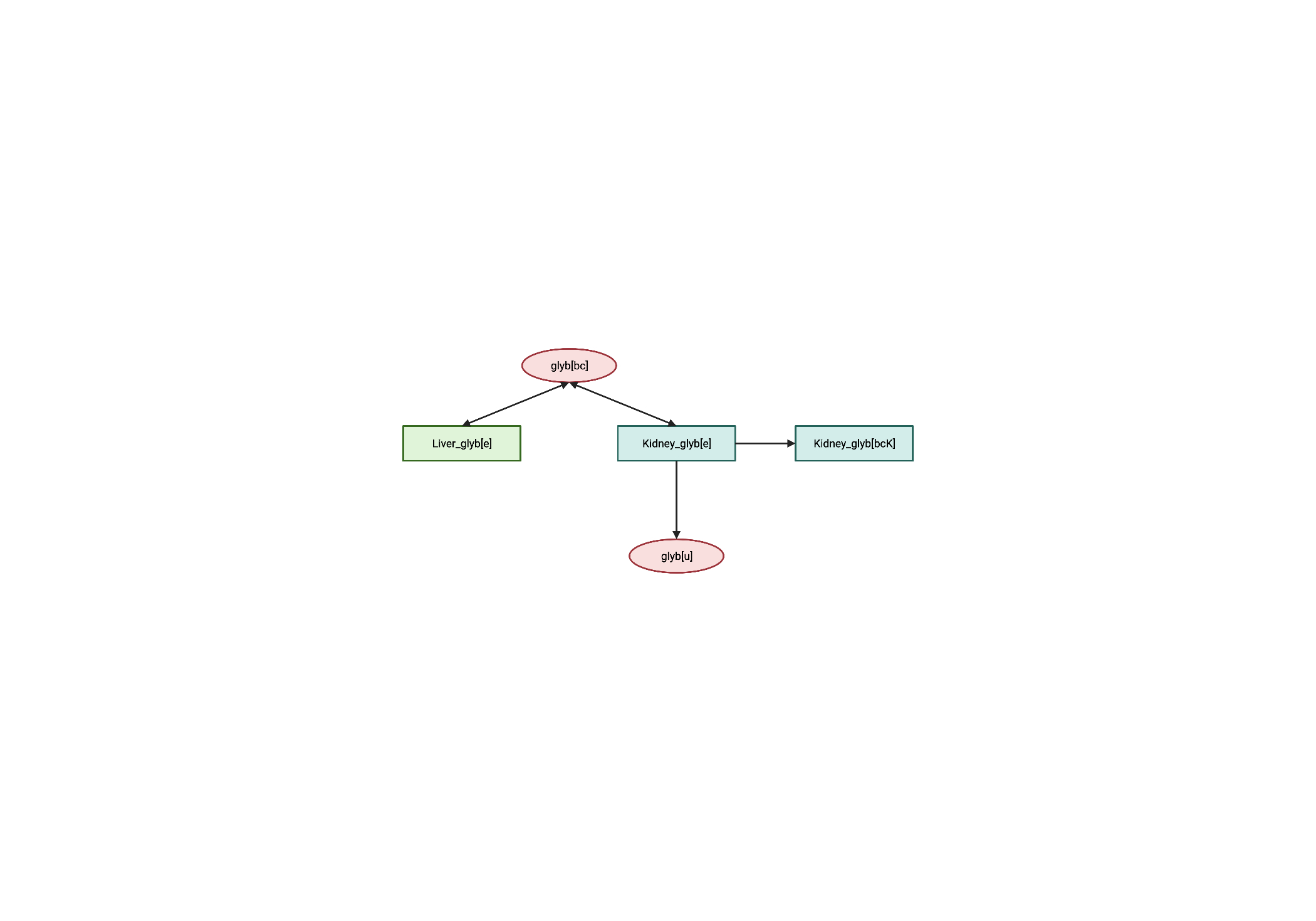

Supplement: Supplementary file 2 [file Image1.JPEG]

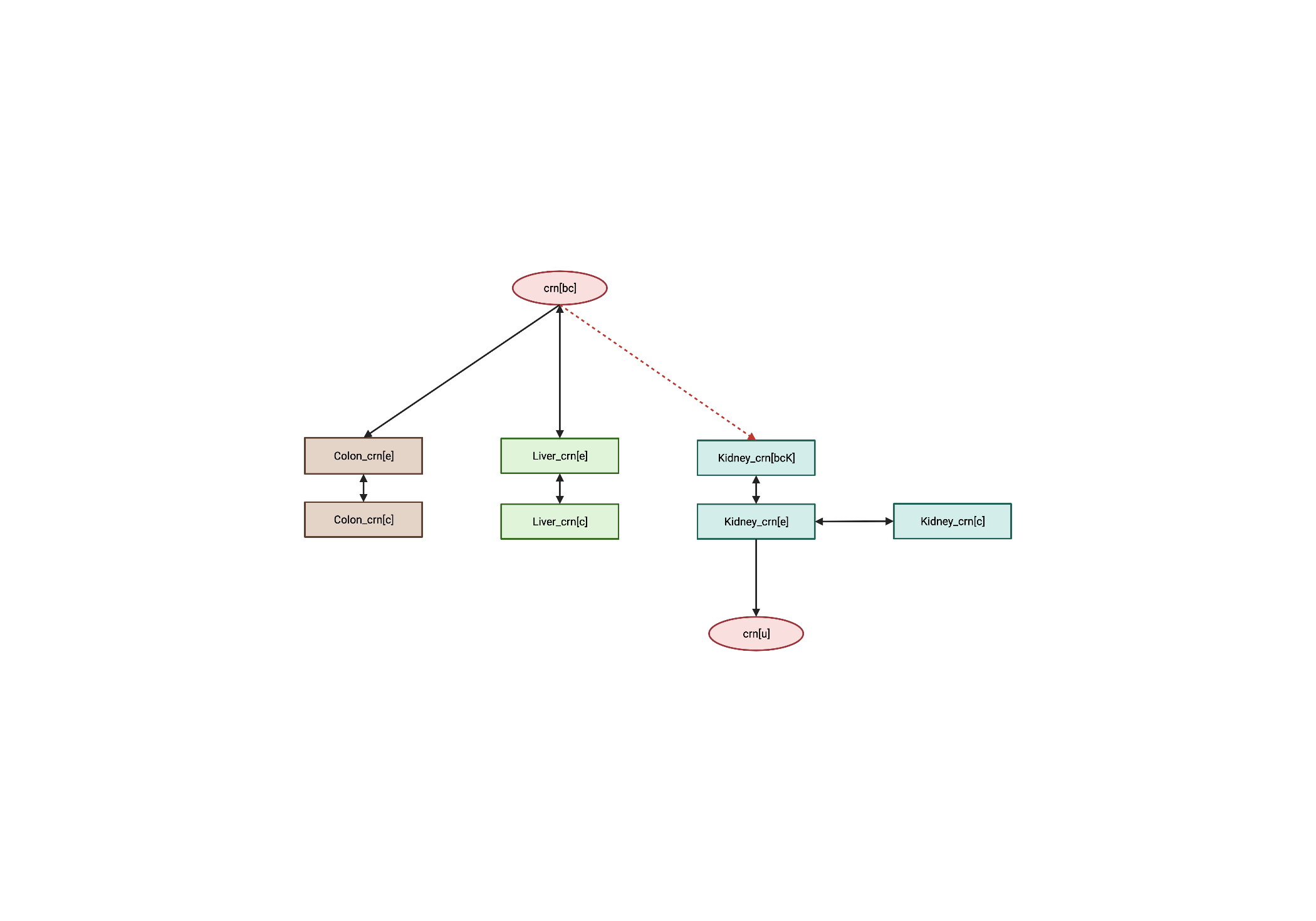

Supplement: Supplementary file 3 [file Image2.JPEG]
